# Peroxisomal Disorders and Their Mouse Models Point to Essential Roles of Peroxisomes for Retinal Integrity

**DOI:** 10.3390/ijms22084101

**Published:** 2021-04-15

**Authors:** Yannick Das, Daniëlle Swinkels, Myriam Baes

**Affiliations:** Lab of Cell Metabolism, Department of Pharmaceutical and Pharmacological Sciences, KU Leuven, 3000 Leuven, Belgium; yannick.das@kuleuven.be (Y.D.); danielle.swinkels@kuleuven.be (D.S.)

**Keywords:** peroxisome, Zellweger, metabolism, fatty acid, retina

## Abstract

Peroxisomes are multifunctional organelles, well known for their role in cellular lipid homeostasis. Their importance is highlighted by the life-threatening diseases caused by peroxisomal dysfunction. Importantly, most patients suffering from peroxisomal biogenesis disorders, even those with a milder disease course, present with a number of ocular symptoms, including retinopathy. Patients with a selective defect in either peroxisomal α- or β-oxidation or ether lipid synthesis also suffer from vision problems. In this review, we thoroughly discuss the ophthalmological pathology in peroxisomal disorder patients and, where possible, the corresponding animal models, with a special emphasis on the retina. In addition, we attempt to link the observed retinal phenotype to the underlying biochemical alterations. It appears that the retinal pathology is highly variable and the lack of histopathological descriptions in patients hampers the translation of the findings in the mouse models. Furthermore, it becomes clear that there are still large gaps in the current knowledge on the contribution of the different metabolic disturbances to the retinopathy, but branched chain fatty acid accumulation and impaired retinal PUFA homeostasis are likely important factors.

## 1. Introduction

Peroxisomes are ubiquitous cellular organelles that host a number of exclusive anabolic and catabolic lipid conversions besides other metabolic and non-metabolic functions. This includes the α-oxidation of phytanic acid, the β-oxidation of various substrates and ether lipid synthesis [1,2].

Optimal peroxisomal function is of pivotal importance for human health, as highlighted by a set of inherited peroxisomal diseases. This group of rare metabolic diseases can be categorized into peroxisomal biogenesis disorders and single enzyme deficiencies. The clinical presentation strongly varies and depends on the involved metabolic pathways and the type of mutation [3]. Interestingly, almost all peroxisomal disorder patients, even those with a mild clinical presentation, develop an ocular phenotype, which often includes retinopathy [4].

Roughly, the retina is subdivided into the neural retina and the retinal pigment epithelium (RPE). The neural retina consists of six distinct layers containing six large groups of specialized cell types: photoreceptors, interneurons (horizontal, amacrine and bipolar cells), ganglion cells, and Müller cells, which are a type of glial cell [5].

The photoreceptors are the light sensitive neurons of the retina. There are two types, rods and cones, that differ in spatial distribution and function. In the human retina, cones are concentrated in the macula, with the highest density in its center (i.e., the fovea), enable day and colour vision and establish high visual acuity. Rods are more prominent in the periphery and are necessary for night and peripheral vision. Both cell types exhibit a similar peculiar morphology and are built up of five main compartments: the outer and inner segment that are connected via the connecting cilium, the cell body containing the nucleus, the inner fiber and synaptic terminal [6].

The photoreceptor outer segment (POS) is a form of modified primary cilium that is filled with hundreds of lipid-rich disc membranes, which contain the light sensitive visual pigments. As elaborated on in sections below, the fatty acid composition is unusual and might require peroxisomal function for their synthesis and/or degradation. The inner segment is important for the synthesis of POS components that are subsequently delivered via the connecting cilium. Lastly, the inner fiber is the axon of the photoreceptor, which makes a synapse with interneurons. The latter relay to the ganglion cells, whose axons form the optic nerve that will transfer the visual signal to the brain [5,7].

The RPE is a monolayer of postmitotic and highly polarized cells that exert an array of functions, with the primary goal to maintain healthy photoreceptors [8,9]. One important characteristic of RPE cells is that they are connected by tight junctions, establishing the outer blood–retina barrier, and they act as important distributors of nutrients and waste products. RPE cells are also essential players in the visual cycle, i.e., the re-isomerization and recycling of 11-*cis*-retinal. In addition, they are involved in the phagocytosis of damaged POS that are shed on a daily basis by photoreceptors. The building blocks are either recycled towards the photoreceptors or degraded. Of note, in the human retina, each RPE cell supports approximately 45 photoreceptors and it takes about 10 days to replace an entire outer segment. This demonstrates that RPE cells are exposed to high amounts of proteins and lipids originating from the POS and, therefore, have to be very active metabolically in order to process the huge amount of cellular material. Hence, it is conceivable that lipid metabolism constitutes an important activity of these cells [8,9]. Finally, it is important to mention that the energy metabolism of photoreceptors and RPE cells is strongly intertwined in a so-called metabolic ecosystem [10].

In this review, we first discuss the distribution of peroxisomes in the retina. Next, we summarize the ophthalmological pathologies in the different peroxisomal disorders in humans and, where applicable, in animal models, with a special focus on the retinal symptoms. Subsequently, the potential role of deregulated lipids as a cause of retinopathy will be scrutinized.

## 2. The Location of Peroxisomes in the Retina

Although peroxisomes reside in all eukaryotic cells, with the exception of red blood cells, their abundance, content and size strongly vary between cell types and according to the environmental conditions [11]. Decades ago, diaminobenzidine (DAB) histochemistry was the golden standard to visualize peroxisomes. This technique detects the peroxidatic activity of catalase, a peroxisomal enzyme involved in redox metabolism. In different species, catalase-positive peroxisomes were detected in the RPE and to a lesser extent in the neural retina, observations that were later confirmed by immunohistochemical stainings (IHC) for catalase [12,13,14,15,16,17].

**Figure 1:**
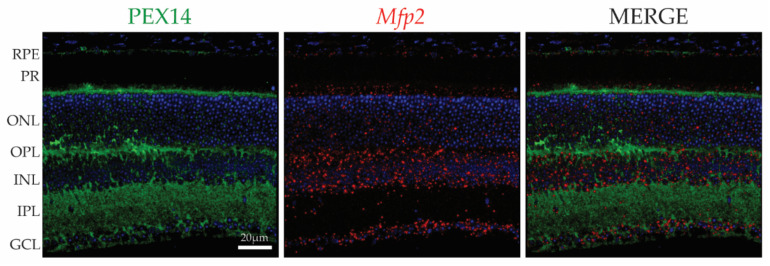
Spatial distribution of PEX14 protein and *Mfp2* mRNA in the mouse retina. IHC for PEX14 (green) and RNAscope^®^ in situ hybridization for *Mfp2* (red) reveals the presence of peroxisomes in the RPE, photoreceptors, interneurons and ganglion cells. Nuclei were counterstained with Hoechst (blue). PEX14, peroxin 14; *Mfp2*, multifunctional protein 2; RPE, retinal pigment epithelium; PR, photoreceptor layer; ONL, outer nuclear layer; OPL, outer plexiform layer; INL, inner nuclear layer; IPL, inner plexiform layer; GCL, ganglion cell layer.

Only recently, more systematic studies were performed to elucidate the distribution of peroxisomes [18]. IHC and immunoblotting for peroxins (such as PEX14, PEX1 and PEX6), which are essential for peroxisome biogenesis and can be considered as housekeeping peroxisomal proteins, revealed their presence in the RPE, photoreceptor inner segments, interneurons and ganglion cells (Figure 1) [18,19,20,21,22]. Transcripts of the central enzyme of peroxisomal β-oxidation, Multifunctional protein 2 (MFP2) showed a similar spatial distribution compared to PEX14 immunoreactivity (Figure 1) [23]. In contrast, other proteins involved in peroxisomal lipid metabolism were differentially expressed between the RPE and neural retina, whereby some were more enriched in the RPE and others in the neural retina [18]. One striking example is the expression pattern of the ATP-binding cassette type D (ABCD) transporters that couple ATP hydrolysis to substrate translocation over the peroxisomal membrane, prior to its degradation. There are three types of ABCD transporters that have different substrate specificities. Although the specificity is not absolute, ABCD1 (adrenoleukodystrophy protein, ALDP) preferentially imports VLCFAs, ABCD2 (adrenoleukodystrophy related protein, ALDRP) transfers PUFAs, whereas ABCD3 (peroxisomal membrane protein 70, PMP70) handles dicarboxylic acids, 2-methyl branched chain fatty acids and bile acid intermediates [24]. ABCD1 was equally expressed in the RPE and neural retina, while ABCD2 and ABCD3 were more abundant in the RPE, suggesting that peroxisomal β-oxidation handles specific substrates in the different retinal cell types [18].

Interestingly, it was reported that peroxisome abundance (visualized by DAB histochemistry coupled to electron microscopy) in the RPE followed the diurnal changes in POS phagocytosis, with higher peroxisome numbers at the burst of phagocytosis [25]. However, these observations were recently refuted as PEX14 and ABCD3 protein levels were relatively constant throughout the day [22]. Still, in the latter report, increased catalase activity coinciding with POS phagocytosis was shown, but a clear link remains to be established.

Altogether, these findings suggest that peroxisomes exert different functions in the inner and outer retina. However, their exact role in retinal homeostasis and integrity has only recently gained more attention.

## 3. Zellweger Spectrum Disorders

Peroxisome biogenesis involves the assembly of peroxisomal membranes and maintenance of functional peroxisomes. The latter includes the import of the matrix proteins, containing a peroxisomal targeting signal (PTS1 or PTS2), from the cytosol. Peroxisome biogenesis requires the close collaboration of the so-called peroxins, encoded by one of fourteen *PEX* genes in mammals. Defective peroxisomal biogenesis results in an array of clinical disease presentations, described as Zellweger spectrum disorders (ZSD), in which the import of both PTS1 and PTS2 proteins is defective, and rhizomelic chondrodysplasia punctata (RCDP) type 1 and 5, in which only PTS2 protein import is hampered [3,26].

To date, mutations in thirteen *PEX* genes are known to cause ZSD, with *PEX1* and *PEX6* mutations being the most prevalent [26,27]. The disease severity depends on the type of mutation and, hence, on the residual function of the corresponding peroxin. Patients suffering from the most severe phenotype of the ZSD, i.e., Zellweger syndrome (also known as cerebro-hepato-renal syndrome), present with general multi-organ failure and mostly die within the first year of life [26].

### 3.1. Retinopathy: A Recurrent Phenomenon

The diverse clinical image of ZSD patients often entails a number of ocular symptoms, even in patients surviving into adulthood. These symptoms include optic atrophy, glaucoma, cataract and nystagmus. In addition, retinopathy is also frequently observed, with abnormal retinal pigmentation, retinal degeneration (i.e., loss of retinal cells) and impaired ERG responses [4,26,28,29,30]. The pigmentary retinopathy has a varied clinical presentation, including retinitis pigmentosa [26]. In the latter, rods are damaged first, resulting in night blindness and peripheral vision loss, followed by loss of cones, leading to complete blindness. The typical fundoscopic image includes retinal vessel attenuation, optic disc pallor and abnormal retinal pigmentation (i.e., either hypopigmentation due to RPE atrophy or hyperpigmentation with bone spicule-shaped deposits), starting in the periphery (cf. rods first affected) and progressing towards the macula (cf. secondary cone damage) [31]. Furthermore, the diverse retinal pathology is underscored by the fact that several patients presenting with a disease phenotype of Leber congenital amaurosis [32,33,34] and Usher syndrome [20,35,36,37], two well-known diseases that are characterized by pigmentary retinopathy, have been re-diagnosed as suffering from ZSD.

Interestingly, the ophthalmological symptoms of patients suffering from Heimler syndrome, the mildest ZSD and caused by mutations in the *PEX1*, *PEX6* or *PEX26* gene, are confined to the retina. The pathology varies from abnormal retinal pigmentation to macular dystrophy. Other symptoms are sensorineural hearing loss, enamel hypoplasia and nail abnormalities (beau’s lines) [38].

Furthermore, it is remarkable that patients with mild mutations in other *PEX* genes, i.e., *PEX2*, *PEX10* and *PEX12*, primarily present with an early-onset cerebellar ataxia phenotype in the absence of retinopathy. Notably, the corresponding peroxins are part of the same complex in the peroxisomal membrane and it is to date unresolved why defects in these peroxins result in a distinct clinical phenotype [26,39].

### 3.2. Histopathology

There are only a few old histopathological descriptions of the retinas of severe Zellweger syndrome patients, reporting RPE atrophy, bi-leaflet inclusions in the RPE, photoreceptor outer and inner segment degeneration, photoreceptor loss, atrophy of the ganglion cells and optic nerve. In addition, macrophage infiltration in the retina was observed [40,41].

Besides a fundoscopic evaluation, the development of the non-invasive technique optical coherence tomography (OCT) has greatly improved the diagnosis of inherited retinal diseases and allows the in vivo assessment of the retinal structure [42]. Fundoscopy of a 20-month-old patient, with a slightly less severe phenotype compared to Zellweger syndrome, revealed normal retinal vessels and optic nerve, but the macula appeared dull without a foveal light reflex and there was abnormal retinal pigmentation in a leopard-spot pattern in the midperiphery. OCT detected severe outer retinal atrophy in both the fovea and the areas with pigmentary changes. In addition, in the latter, hyperreflective spots on the RPE and bulging into the POS layer were observed. Of note, this patient also displayed severely reduced dark- and light-adapted ERG responses, indicating impaired rod and cone function, which is in accordance with the observed structural anomalies [29].

The retinal structure of two mild *PEX1* patients was described. These patients were initially suspected to suffer from Usher syndrome [37] and Leber congenital amaurosis [33]. The alleged Usher syndrome patient developed neurosensory hearing loss at the age of 3 months and night-blindness at the age of 9 years. Fundoscopy and OCT revealed RPE and macular dystrophy complicated by macular cystoid oedema. Dark-adapted ERG responses were strongly impaired while light-adapted ERG was only mildly affected [37]. The retinal pathology of the re-diagnosed Leber congenital amaurosis patient appeared more complicated, as the macula and periphery were affected, while the perifoveal region was not. Fundoscopy and OCT showed extensive RPE atrophy in the affected regions and severe foveal thinning, respectively. In addition, no ERG responses were detectable [33].

Finally, several reports described the OCT findings in Heimler syndrome patients. Outer retinal pathology, mostly complicated by cystoid macular oedema, was frequently observed, involving photoreceptor abnormalities (e.g., POS disruption and loss of the junction between the photoreceptor outer and inner segments), photoreceptor loss and RPE atrophy [21,43,44,45,46].

Together, these pathological investigations indicate that loss of peroxisome function induces photoreceptor degeneration, but also the RPE and the inner retina are affected.

### 3.3. Insights form the Pex1 Knock-In Mouse Model

While several mouse models for ZSD have been developed, only the retinal phenotype of the *Pex1* knock-in mouse, homozygous for the most common mild human *PEX1* mutation (G843D), was reported [19,47].

Adult mice displayed reduced visual acuity. However, the onset of the pathology occurred already in the juvenile stage, involving both photoreceptors and interneurons. Although no photoreceptor loss was detected, rods and cones displayed distinct abnormalities. At the age of 2 weeks, the photoreceptor outer and inner segments were decreased in length, but this normalized by 3 weeks of age. In addition, rod function, measured via ERG (i.e., scotopic a-wave), was decreased in the juvenile mice, then normalized at the age of 4 weeks and subsequently gradually declined. It should be noted that the *Pex1* knock-in mice are severely growth impaired compared to age matched controls during the lactation period, possibly also resulting in a delay in photoreceptor maturation.

Cone function (i.e., photopic b-wave) was already impaired at the age of 2 weeks and remained affected, which is in contrast to rods. Moreover, IHC for cone arrestin, labelling cones from the POS to the synaptic terminals, showed loss of outer and inner segments and of connections with the interneurons. These morphological changes were detected at the age of 6 weeks with no further information at earlier time points. At the ultrastructural level, photoreceptors, presumably rods, of 32-week-old mice showed normal POS, but disorganized inner segments. The exact consequence of the latter is unclear, since the main function of the inner segments is to synthesize the outer segments.

ERG analyses also revealed impaired function of bipolar cells (i.e., reduced scotopic b-wave) from the juvenile period into adulthood. Interestingly, the functional deficits preceded the morphological defects, which were only shown in 32-week-old mice via IHC for protein kinase C alpha (PKCα). Markers of other interneurons seemed to be unaltered. The ongoing photoreceptor stress might induce an adaptive response in the underlying retinal cells, which is part of a process called retinal remodelling [48]. On the other hand, in view of the abundance of peroxisomes in interneurons, a primary defect caused by peroxisomal dysfunction in these cells cannot be excluded and requires further research.

Finally, transmission of the visual signal to the brain was also affected, as shown via measurements of the visual evoked potentials. This could coincide with the retinal phenotype, but the optic nerve and visual cortex were not investigated and defects at these levels cannot be ruled out.

Hence, it appears that some of the retinal pathologies observed in peroxisome biogenesis disorders are recapitulated in the *Pex1* knock-in mice, including photoreceptor dysfunction, but, in contrast to the patients, anomalies of the RPE were not reported in the mice.

## 4. Single Enzyme Deficiencies

In peroxisomal biogenesis disorders, all metabolic pathways are dysfunctional, obscuring the cause of the retinal pathology. More information on the metabolic origin can be obtained by examining peroxisomal single enzyme deficiencies in which only one metabolic path is defective. An overview of the metabolic abnormalities and the ocular pathology in peroxisomal biogenesis disorders and single enzyme deficiencies is provided in Table 1.

### 4.1. Defects in α-Oxidation

#### 4.1.1. Retinitis Pigmentosa in Refsum Disease

Patients with a defect in peroxisomal α-oxidation are diagnosed with (adult) Refsum disease. To date, mutations in two genes have been identified to lie at the basis of the disease. The most frequent cause is a mutation in the gene encoding phytanoyl-CoA hydroxylase (PHYH) that catalyses the first reaction of the α-oxidation pathway. Mild mutations in the *PEX7* gene are the second cause. The corresponding peroxin is dedicated to the import of PTS2-containing proteins, i.e., alkylglycerone phosphate synthase (AGPS), PHYH and acetyl-CoA acyltransferase (ACAA1) [3,49]. Although in this case, the disease is in principle a peroxisomal biogenesis disorder, mild mutations lead to the impaired function of PHYH, while plasmalogen levels are either near normal or sufficient to prevent associated pathologies. Hence, the phenotype corresponds to a selective defect in peroxisomal α-oxidation [86,87].

**Figure 2:**
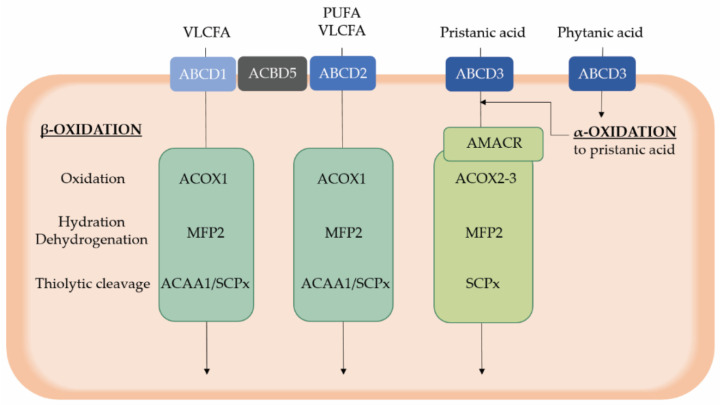
Overview of peroxisomal α- and β-oxidation of substrates relevant for retinal homeostasis. Phytanic acid is catabolized via peroxisomal α-oxidation, resulting in the formation of the 2-methyl branched chain fatty acid pristanic acid that subsequently undergoes peroxisomal β-oxidation similar to other substrates, including VLCFAs and PUFAs. After activation to a CoA ester, substrates are translocated in peroxisomes via three ABCD transporters, which exert a differential, although not absolute, substrate specificity. Peroxisomal β-oxidation occurs in four steps whereby the second and third step are catalyzed by a multifunctional protein. Of note, pristanic acid first needs to be converted into the S-configuration by AMACR. Furthermore, ACBD5 is assumed to present VLCFAs to the ABCD transporters, besides its role in the tethering of peroxisomes to the endoplasmic reticulum. ABCD, ATP binding cassette subfamily D; ACAA1, acetyl-CoA acyltransferase 1; ACBD5, Acyl-CoA binding domain containing protein 5; ACOX, acyl-CoA oxidase; D/THCA, di- and trihydroxycholestanoic acid; MFP, multifunctional protein; PUFAs, polyunsaturated fatty acids; SCPx, sterol carrier protein x; VLCFAs, very long chain fatty acids.

Clinically, retinitis pigmentosa is considered as the cardinal symptom, besides the polyneuropathy. Other frequently observed ophthalmological symptoms are miosis, attenuated pupillary light responses, iris atrophy, and cataract. Biochemically, patients are characterized by phytanic acid accumulation [49]. This long chain branched fatty acid is catabolized by peroxisomes via α-oxidation to produce pristanic acid, which in turn undergoes peroxisomal β-oxidation (Figure 2) [2]. Phytanic acid is a metabolic by-product of chlorophyll, produced by the intestinal flora of ruminants, and the main sources are the dietary uptake from dairy products and the meat of those animals [49].

Although the retinal deterioration is halted by restricted dietary intake of phytanic acid, which is compelling evidence that the increased levels are the disease-causing factor, the underlying mechanisms for the impaired photoreceptor survival are still elusive. Historically, two hypotheses were postulated, i.e., the antimetabolite and the molecular distortion hypothesis [49,88]. The first one claims that phytanic acid hampers the regeneration of 11-*cis*-retinal, while the molecular distortion hypothesis states that phytanic acid negatively alters the membrane composition and structure [88,89]. In addition, more recently, several other possible mechanisms have been postulated, such as mitochondrial toxicity [39].

#### 4.1.2. Insights from In Vivo and In Vitro Models

To investigate the effects of increased phytanic acid levels, several animal models have been established, but a retinal phenotype similar to the human situation was not mimicked. Firstly, retinas of rats fed a phytanic acid-rich diet did not show abnormal ERG responses nor retinal degeneration [90]. Secondly, the short-term pathological effects of phytanic acid accumulation in a mouse model for Refsum disease were investigated. Hereto, PHYH deficient mice were fed a phytol-rich diet. The mice did not develop gross eye abnormalities upon microscopic investigation, although possible retinopathy was not thoroughly investigated. Therefore, it cannot be ruled out that retinal function is affected and further research is needed [91].

The impact of phytanic acid on RPE cells was studied in vitro by Bernstein et al. [88]. Hereto, both primary human and bovine RPE cells were exposed to elevated phytanic acid levels and morphological changes were investigated via electron microscopy. The changes included RPE swelling, development of presumably lipid-filled vacuoles, ER dissolution, and loss of apical microvilli, but mitochondria were not affected. Moreover, human RPE cells were more sensitive to elevated phytanic acid levels than bovine RPE cells and effects of moderately elevated levels were reversible [88].

### 4.2. Peroxisomal β-Oxidation Deficiency

Peroxisomal β-oxidation consists of a four-step process (i.e., oxidation, hydration, dehydrogenation, and thiolytic cleavage) and metabolizes a broad set of substrates, serving both catabolic and anabolic purposes (Figure 2). More specifically, very long chain (polyunsaturated) fatty acids (VLC-(PU)FAs; > C22), the 2-methyl branched chain fatty acid pristanic acid, dicarboxylic fatty acids and eicosanoid inflammatory mediators exclusively depend on peroxisomal β-oxidation for their breakdown. Although it was shown that peroxisomes are also able to oxidize short, medium, and long chain fatty acids (<C20), these are preferentially metabolized in mitochondria [2,92]. In addition, the bile acid intermediates dihydroxycholestanoic acid (DHCA) and trihydroxycholestanoic acid (THCA) undergo one round of peroxisomal β-oxidation in the process to form mature bile acids in the liver [2].

**Figure 3:**
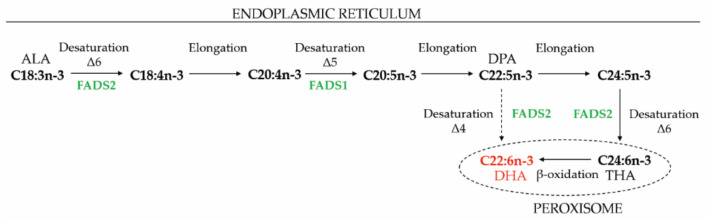
De novo biosynthesis of DHA. ALA is elongated and desaturated to THA in the ER and subsequently retroconverted to DHA via one cycle of peroxisomal β-oxidation (also known as the “Sprecher shunt”). However, whereas it was previously thought that DPA cannot be directly converted to DHA, FADS2 activity on DPA was recently shown [99], but the contribution to DHA synthesis in different cell types has not been resolved. ALA, α-linolenic acid; DHA, docosahexaenoic acid; DPA, docosapentaenoic acid; FADS, fatty acid desaturase; THA, tetrahexaenoic acid.

Peroxisomal β-oxidation executes a dual function in long chain PUFA metabolism, i.e., synthesis and degradation [2]. This is particularly interesting with respect to the specific lipid profile of the retina, which is highly enriched in PUFAs. Firstly, peroxisomal β-oxidation is essential for the synthesis of the *n*-3 PUFA docosahexaenoic acid (DHA, C22:6*n*-3) that is the most abundant PUFA in the POS. Hereto, the essential PUFA α-linolenic acid (C18:3*n*-3) undergoes several sequential elongation and desaturation reactions in the ER, leading to the formation of tetracosahexaenoic acid (C24:6*n*-3), which is subsequently oxidized to produce DHA (i.e., retroconversion to DHA). This pathway, also known as the “Sprecher shunt”, involves one cycle of peroxisomal β-oxidation (Figure 3) [93,94]. This DHA synthesis pathway was discovered in the liver, but it was shown that both RPE cells and photoreceptors are able to execute these reactions, albeit to a lesser extent [95,96,97,98]. Of note, the DHA-related PUFA in the *n*-6 series, i.e., C22:5*n*-6, undergoes similar reactions, starting from linoleic acid (C18:2*n*-6). Secondly, for their degradation, the long chain PUFAs also require β-oxidation. However, the contribution of peroxisomal versus mitochondrial β-oxidation was not fully elucidated and this may be species and cell type dependent [2]

To date, defects in seven proteins involved in the different steps of peroxisomal β-oxidation have been identified and include (i) the ABCD1 transporter, (ii) multifunctional protein 2 (MFP2), (iii) acyl-CoA oxidase 1 (ACOX1), (iv) α-methylacyl-CoA racemase (AMACR), (v) acyl-CoA oxidase 2 (ACOX2), (vi) the ABCD3 transporter and (vii) sterol carrier protein x (SCPx), in decreasing order of occurrence [39]. Recently, Ferdinandusse et al. proposed to classify acyl-CoA binding domain containing protein 5 (ACBD5) deficiency in this subgroup, although ACBD5 does not participate in the peroxisomal β-oxidation cycle [81].

#### 4.2.1. X-Linked Adrenoleukodystrophy

X-linked adrenoleukodystrophy (X-ALD) is caused by mutations in the *ABCD1* gene. This leads to the accumulation of saturated VLCFAs, due to their impaired peroxisomal translocation preceding the degradation via β-oxidation. Even though patients can be asymptomatic or present with an isolated adrenocortical insufficiency (i.e., Addison-only phenotype), the two main disease presentations are adrenomyeloneuropathy (AMN) and cerebral ALD (CALD). AMN is mainly characterized by a progressive, non-inflammatory axonal polyneuropathy in the spinal cord, whereas CALD involves inflammatory cerebral demyelination [50,100].

The neurological decline in CALD also involves a decrease in visual acuity and development of visual field defects, cortical blindness and eye motility problems (such as strabismus). These symptoms are caused by extensive brain lesions and demyelination of the visual tract, coinciding with optic disc pallor upon fundoscopic examination [50,51,52,53]. In addition, via OCT, retinal nerve fiber layer thinning and ganglion cell loss were detected that were most pronounced in the macular region. Importantly, in contrast to other peroxisomal β-oxidation disorders, no pathology was seen in the outer retina [52,54].

#### 4.2.2. Acyl-CoA Oxidase 1 Deficiency

ACOX1 catalyses the first desaturation step of the peroxisomal β-oxidation and is assumed to only play a role in the degradation of straight chains, including saturated and polyunsaturated VLCFAs [2]. Severely affected ACOX1 deficiency patients are indistinguishable from those suffering from Zellweger syndrome. Likewise, similar (early-onset) ocular symptoms are observed, including abnormal retinal pigmentation, retinal degeneration and optic atrophy [65,66,67,68,69,70,71].

Although most patients do not survive into adolescence, two siblings in their 50s and with a milder disease course were identified. Interestingly, fundoscopic analysis uncovered pronounced ophthalmological pathology, including retinitis pigmentosa and small lens opacities, but sparing of the optic nerve in the male patient. Unfortunately, no information on the retinal involvement in his sister was provided, due to the presence of bilateral cataracts obscuring the fundi [72].

Already more than 20 years ago, *Acox1* knockout mice were generated [101], but the ocular phenotype was not investigated.

#### 4.2.3. Multifunctional Protein 2 Deficiency

MFP2 executes the second (hydration) and third (dehydrogenation) step and displays a broad substrate specificity [2]. This leads to accumulation of VLCFAs, the branched chain fatty acid pristanic acid and bile acid intermediates and shortage of DHA in MFP2 deficient patients. Like ACOX1 deficiency, the clinical presentation of severely affected patients resembles that of Zellweger syndrome, including similar ocular symptoms [76]. More recently, MFP2 deficient patients with a milder disease phenotype with juvenile onset were described. Interestingly, despite normal visual acuity, abnormal retinal pigmentation was reported [77,78,79].

To study the mechanisms underlying the disease course of MFP2 deficiency, our laboratory generated global MFP2 knockout mice (*Mfp2^−/−^* mice) [102]. Hereto, the first three exons of the *Hsd17b4* gene were deleted, resulting in the loss of both the hydratase and dehydrogenase activity. These mice displayed decreased retinal function, already at the age of 3 weeks, and reduced visual acuity. Moreover, we observed a dual phenotype, in which photoreceptors and RPE cells were severely affected [23]. On one hand, MFP2 deficiency caused impaired photoreceptor development, and more specifically impaired maturation, resulting in shortened outer and inner segments with surprisingly normal ultrastructure at the age of 3 weeks. It is noteworthy that this decrease in photoreceptor length persisted and even worsened upon aging, which is in contrast to the *Pex1* knock-in mice. Together with this morphological abnormality, *Mfp2^−/−^* retinas exhibited severe changes in gene expression in almost all retinal cells, but especially in rods and cones (e.g., downregulation of genes involved in phototransduction) and in retinal glia cells (i.e., increase in inflammation related processes). The latter coincides with microglial activation and migration towards the RPE, shown via IHC for ionized calcium binding adaptor molecule 1 (Iba1). On the other hand, we also observed a degenerative component in the retinal phenotype of the *Mfp2^−/−^* mice. At the age of 9 weeks, we observed a progressive loss of photoreceptors, which was already initiated at the age of 3 weeks as shown by a marked increase in TUNEL positive photoreceptor nuclei. Moreover, RPE cells seemed to invade the POS layer. Furthermore, we performed in-depth lipid analyses on the retinas of *Mfp2^−/−^* mice, as a first approach to elucidate the mechanisms underlying the retinal pathology in peroxisomal β-oxidation deficiency. In Section 6, these results will be further discussed and matched to information from the patients. Since photoreceptors and RPE are strongly interdependent, the cause-consequence relationship of the degeneration of the outer retina still needs to be elucidated. Furthermore, in view of the abundance of *Mfp2* transcripts in the inner retina, the impaired b-waves and altered transcripts of inner retinal cells (amacrine, bipolar and ganglion cells), this also deserves further research.

Unfortunately, it remains elusive to what extent the retinal pathology of the *Mfp2^−/−^* mice recapitulates that of the patients. To our knowledge, no histopathological descriptions of the retinas of MFP2 deficient patients were reported. There are, however, several similarities with the histopathological changes observed in the abovementioned Zellweger syndrome patients. Indeed, photoreceptor loss and infiltration of inflammatory cells in the retina of *PEX* patients was described and the RPE atrophy and protrusion of RPE cells into the POS layer of *Mfp2^−/−^* mice could correspond with the hyper-reflexive spots under the RPE upon OCT.

#### 4.2.4. Deficient Oxidation of Branched Chain Fatty Acids and Bile Acid Synthesis

The branched chain fatty acid pristanic acid and the bile acid intermediates DHCA and THCA are imported into peroxisomes by the ABCD3 transporter [24]. Prior to their metabolism via β-oxidation by ACOX2 (pristanic acid and bile acids) or ACOX3 (pristanic acid) and subsequently by MFP2 and SCPx, the substrates are converted into their *S*-configuration by α-methylacyl-CoA racemase (AMACR) (Figure 2) [2]. Although these proteins are involved in the metabolism of the same substrates, the metabolic abnormalities and clinical presentation differ profoundly.

Firstly, AMACR deficiency is biochemically characterized by the accumulation of pristanic acid, DHCA and THCA. Clinically, one patient has been described with severe early-onset liver dysfunction [103], while a dozen other patients present with a late-onset neuropathy, with symptoms resembling Refsum disease, and no liver dysfunction. Importantly, the majority presents with pigmentary retinopathy, including retinitis pigmentosa, and impaired visual acuity. Additional ocular symptoms in some patients are optic atrophy, cataract and visual field defects [56,57,58,59,60,61,62,63,64]. The retina of the AMACR knockout mouse has not been investigated [104].

Given the similarity of the retinal pathology of AMACR deficiency with Refsum disease, the metabolic cause is likely the accumulation of the branched chain fatty acid pristanic acid. In this respect, it is quite surprising that the only described SCPx deficient patient does not show ocular symptoms. Yet, he had elevated pristanic acid levels and displayed profound neurological symptoms [80].

Furthermore, the few ABCD3 and ACOX2 deficient patients are characterized by relatively normal pristanic acid but elevated DHCA and THCA levels. Their clinical symptoms originate from primary liver dysfunction, and similar to the SCPx patient, no ophthalmological abnormalities were reported [55,73,74,75]. However, most of these patients were described at an early age, and it cannot be excluded that retinopathy develops later.

#### 4.2.5. Acyl-CoA Binding Domain Containing Protein 5 Deficiency

ACBD5 is a quite recently described tail-anchored peroxisomal membrane protein that was assigned with multiple functions. Its main function is being part of a tethering complex that establishes contacts between peroxisomes and the endoplasmic reticulum (ER) [105,106,107]. The name refers to an acyl-CoA binding domain and it was postulated to present VLCFAs to the ABCD1 transporter [81,108]. ACBD5 deficiency was first described in three siblings, by using exome sequencing on a large cohort of retinopathy patients. Besides the pronounced retinal dystrophy (cone-rod dystrophy), the patients presented with severe white matter disease and spastic paraparesis [82]. Subsequently, Ferdinandusse et al. described another ACBD5 deficient patient with similar clinical symptoms [81]. Analogous to X-ALD patients, the only metabolic abnormality thus far identified in plasma was the increased levels of saturated VLCFAs, which was the reason why Ferdinandusse et al. proposed to categorize ACBD5 deficiency in the group of the peroxisomal β-oxidation deficiencies [81,108]. More recently, an additional ACBD5 deficient patient was identified, in which ophthalmological problems were the first symptoms, followed by mental and motor deterioration [83]. The retinal pathology involved cone-rod dystrophy, diagnosed by ERG analysis. In addition, fundoscopy revealed optic nerve pallor, attenuation of blood vessels and diffuse granularity of the RPE, symptoms also observed in ZSD, and ACOX1 and MFP2 deficiency.

Recently, the initial characterization of the retinal pathology of an ACBD5 deficient mouse model was described, consisting of loss of photoreceptors and microglial and astrocyte activation at the age of 1 year, but a more thorough investigation is warranted [109].

## 5. Ether Phospholipid Deficiency

### 5.1. Rhizomelic Chondrodysplasia Punctata

To date, five types of the disease rhizomelic chondrodysplasia punctata (RCDP) are identified, with mutations in five different genes. Type 1 and 5 are considered as peroxisomal biogenesis disorders leading to impaired import of PTS2 proteins due to mutations in *PEX7* and in exon 9 of *PEX5*, respectively. Type 2, 3, and 4 belong to the single enzyme deficiencies, due to mutations in the *GNPAT*, *AGPS*, and *FAR1* genes [110]. These mutations lead to a selective deficiency in the synthesis of ether phospholipids (also known as plasmanyl phospholipids) that represent a glycerophospholipid subclass in which the fatty acid at the *sn*-1 position is replaced by a long chain fatty alcohol, establishing the characteristic ether bond. Moreover, plasmalogens (also known as plasmenyl phospholipids) are the most abundant ether lipid subspecies, containing a vinyl-ether bond. Biosynthesis of these glycerophospholipid species requires the cooperation of peroxisomes and the ER. The first two steps are executed in the peroxisome by glycerone-phosphate O-acyltransferase (GNPAT) and AGPS. In addition, at the cytosolic side of the peroxisomal membrane, the enzyme fatty acyl-CoA reductase 1 (FAR1) produces the fatty alcohol via the reduction of the corresponding fatty acyl-CoA [84,111].

Besides characteristic bone malformations, impaired growth and a range of neurological symptoms, congenital cataract is a cardinal symptom in RCDP patients. Although plasmalogens are also important constituents of the retina, it seems to be spared [84,85].

### 5.2. Insights from the Different Mouse Models

Several mouse models with deficient ether phospholipid biosynthesis (*Pex7* knockout [112] and hypomorphic [113] mouse, *Gnpat* knockout mouse [114], blind sterile 2 mouse with a spontaneous mutation in *Agps* [115] and two different *Agps* knockout mice [116]) were reported. All mouse models develop bilateral cataract, even those with residual ether phospholipid synthesis. Although lens histology is described in detail in most models, only in the hypomorphic *Pex7* mouse and *Gnpat* knockout mouse the retinal structure was mentioned. The retina of the hypomorphic *Pex7* mouse did not show any gross morphologic alterations [113]. In contrast, retinal abnormalities were reported in the *Gnpat* knockout mouse. Hereby, RPE abnormalities, like vacuolation, hypo- and hyperplasia, pigmentation abnormalities, and accumulation of photoreceptor degradation products were observed in addition to Bruch’s membrane thickening and optic nerve hypoplasia. The *Gnpat* knockout mouse also displayed retinal vasculature abnormalities, in which a persistent hyaloid artery was reported. During development, this artery, extending from the optic disc, provides nutrients to the lens and should eventually completely regress as the retinal vasculature matures [114]. Later, it was elucidated that in this mouse model, the ether phospholipid deficiency interferes with the different stages of retinal vascularization, resulting in a disorganized and dysfunctional network in the mature retina [117]. As there are, to our knowledge, no reports of retinal abnormalities in RCDP patients, it is unclear how to translate the observations from the *Gnpat* knockout mice.

## 6. Candidate Metabolites Causing Retinopathy

Combining the data from the different patient groups, it emerges that retinopathy is a frequent pathology. It appears that ether lipid deficiency does not have a detrimental impact on the retina, but the metabolic origin of the retinopathy in peroxisomal α- and β-oxidation deficient patients seems to be diverse. Recently, we performed in-depth lipid analyses on retinas of *Mfp2^−/−^* mice, as a first approach to elucidate the mechanisms underlying the retinal pathology in peroxisomal β-oxidation deficiency [23]. This was the first time that in a mouse model mimicking peroxisomal dysfunction the retinal lipid composition was associated with the retinal pathology. Given the broad substrate specificity of MFP2, it is instrumental to discuss the biochemical alterations and to put them into context of the retinal pathology in patients with deficient peroxisomal α- and β-oxidation. Hereby, several candidate metabolites deserve a more thorough discussion, including saturated VLCFAs, branched chain fatty acids, DHA and VLC-PUFAs.

### 6.1. Saturated Very Long Chain Fatty Acids

A first metabolic disturbance worth considering is the vast accumulation of saturated VLCFAs in *Mfp2^−/−^* retinas. We found for example a severe increase in the disease marker 1-hexacosanoyl-2-hydroxy-sn-glycero-3-phosphocholine (Lyso-PC 26:0) [118]. However, their involvement in the observed retinal anomalies is questionable because the outer retina in ABCD1 deficient patients, characterized by the accumulation of VLCFAs as sole metabolic abnormality, is always spared [52,54].

### 6.2. Branched Chain Fatty Acids

MFP2 is also involved in the metabolism of the branched chain fatty acid pristanic acid that is formed by the α-oxidation of phytanic acid [2]. It is quite clear that elevated levels of branched chain fatty acids are detrimental for the human retina (cf. Refsum disease and AMACR deficiency), but the situation for the rodent retina is less evident. Rats fed a phytol-rich diet did not develop retinopathy, while the retina of mice with impaired branched chain fatty acid metabolism remains understudied [90,91,104].

Phytanic and pristanic acid in the plasma and brain of *Mfp2^−/−^* mice fed a standard rodent chow were slightly elevated compared to wild type mice, but the levels in plasma remain two orders of magnitude below the levels in Refsum disease or ZSD patients [119]. Although we did not measure branched chain fatty acid levels in the *Mfp2^−/−^* retinas, we hypothesize that their contribution to the retinal degeneration will be limited, but feeding a phytol rich diet could underscore this assumption.

### 6.3. Polyunsaturated Fatty Acids

As already mentioned, the fatty acid composition of POS phospholipids diverges from other membranes, being highly enriched in PUFAs. The most abundant PUFA is docosahexaenoic acid (DHA, C22:6*n*-3), an *n*-3 PUFA that amounts up to approximately 50% of the POS phospholipid fatty acid side chains [120,121,122]. Notably, in some phospholipid species DHA occurs in both the *sn*-1 and *sn*-2 position [123]. Even more exceptional is the relative enrichment in phospholipids containing very long chain polyunsaturated fatty acids (VLC-PUFAs, >C30) in the *sn*-1 and DHA in the *sn*-2 position [124,125,126]. It is noteworthy that cones contain significantly less DHA and VLC-PUFAs than rods (approximately 2-fold). It is, therefore, postulated that cones and cone signalling might have other lipid requirements [127].

#### 6.3.1. Docosahexaenoic Acid

The two main retinal DHA sources are the diet (e.g., fatty fish) and de novo biosynthesis starting from α-linolenic acid (C18:3*n*-3) [128]. The latter occurs primarily in liver and involves one cycle of peroxisomal β-oxidation (Figure 3) [94]. Already, in the 1970s, it was shown that DHA deficiency has a negative impact on retinal function. More specifically, DHA deficient rats displayed reduced ERG responses that could be restored by DHA replenishment [129,130]. The observations of decreased visual function were confirmed in other species, including mouse [131], guinea pig [132,133], rhesus monkeys [134,135,136] and humans (neonates) [137,138,139]. Moreover, supplementing preterm infants with fish oil, generally rich in DHA and eicosapentaenoic acid (EPA, C20:5*n*-3), improved the development of visual function [140].

In view of the presumed role of peroxisomal β-oxidation in PUFA homeostasis and the importance of DHA for retinal function, it was hypothesized that reduced retinal DHA levels lie at the basis of the retinopathy in ZSD and MFP2 deficient patients [141]. Reduced DHA levels are indeed frequently observed in these patients [76,141]. Moreover, in one Zellweger syndrome patient retinal lipids were analysed and a shortage of DHA was found [141]. However, it should be noted that not all MFP2 deficient patients that present with retinopathy display reduced plasma DHA levels, obscuring the possible link between DHA deficiency and retinopathy [76].

In this respect, DHA supplementation to prevent deterioration of visual acuity seemed a plausible treatment option, but two different clinical trials produced contradictory results. More specifically, an open trial showed promising stabilization of the deteriorating retinal function in mildly affected patients [30], while a randomized, double blind and placebo-controlled clinical trial detected no effect of DHA treatment on retinal function [142]. Possibly, the retinal deterioration cannot be halted when it is already ongoing and DHA supplementation should, therefore, start as soon as possible.

#### 6.3.2. Very Long Chain Polyunsaturated Fatty Acids

Synthesis of VLC-PUFAs with more than 26 carbons is mediated by the enzyme elongation of very long chain fatty acids-4 (ELOVL4) in the ER. This enzyme is only found in tissues that contain VLCFAs and VLC-PUFAs with >26 carbons (retina, lens, brain, testis and skin), which implies that the latter are locally produced. In the retina, ELOVL4 is mainly found in the photoreceptor inner segments [126,143]. Although both *n*-3 and *n*-6 fatty acids can be used as elongation substrates, it was shown that EPA is preferentially elongated over arachidonic acid (AA, C20:4*n*-6) and DHA (EPA > AA > DHA) [144,145]. However, this observation has to be put into context. The DHA pool is much larger compared to the EPA and AA pool (±10-fold), and, although it was speculated that DHA is mainly esterified, there is still a considerable amount that is elongated into VLC-PUFAs. In addition, if EPA is indeed the preferred substrate for elongation in the inner segments, it has to be considered that its level in the circulation is very low (Das Y., Vaz F. and Baes M., unpublished observations). In this respect, it can be speculated that the more abundant DHA first undergoes one cycle of peroxisomal β-oxidation and subsequently a desaturation to produce EPA that is further elongated. This could possibly be an additional role of peroxisomes in the photoreceptors, besides local DHA synthesis. After elongation, VLC-PUFAs are esterified to form phospholipids that are mostly integrated into the disc membranes, although it was shown that they are also localised in the synapses [124,146].

The importance of VLC-PUFAs is highlighted by the discovery of mutations in the *ELOVL4* gene, causing autosomal dominant Stargardt 3 Macular Dystrophy (STGD3). Most mutations lead to the synthesis of a truncated and mislocalized protein [143]. In addition to macular photoreceptor degeneration, STGD3 is typically characterized with optic nerve pallor and well-circumscribed macular RPE and choriocapillaris atrophy surrounded by yellow flecks upon fundoscopic examination [126]. Although, to date, the exact mechanism of photoreceptor death in STGD3 still remains elusive, three possible mechanisms are postulated: (i) VLC-PUFA reduction affects photoreceptor function and structure, (ii) the mislocalized truncated protein causes cellular stress, (iii) toxic molecules are generated by the limited function of the truncated protein [143,147]. In line with the first mechanism, STGD3 patients with *ELOVL4*-loss-of-function mutations have been described, which underscores the essential role of VLC-PUFAs in the retina [143].

In addition to the synthesis, it can be predicted that peroxisomal β-oxidation is required for the breakdown of VLC-PUFAs, as this is the only pathway that can handle such long carboxylate chains. The retinal VLC-PUFA status in ZSD and peroxisomal β-oxidation deficiency patients has, however, never been investigated. In the brains of Zellweger syndrome patients, an accumulation of VLC-PUFAs was reported [148,149,150]. More recently, increased levels of VLC-PUFAs were also found in fibroblasts from an ACBD5 deficient patient, but not in X-ALD fibroblasts [108]. These observations lead to the assumption that the ER and peroxisomes need to work together to set a balance between VLC-PUFA synthesis and catabolism.

#### 6.3.3. Retinal PUFA Status of *Mfp2^−/−^* Mice

**Figure 4:**
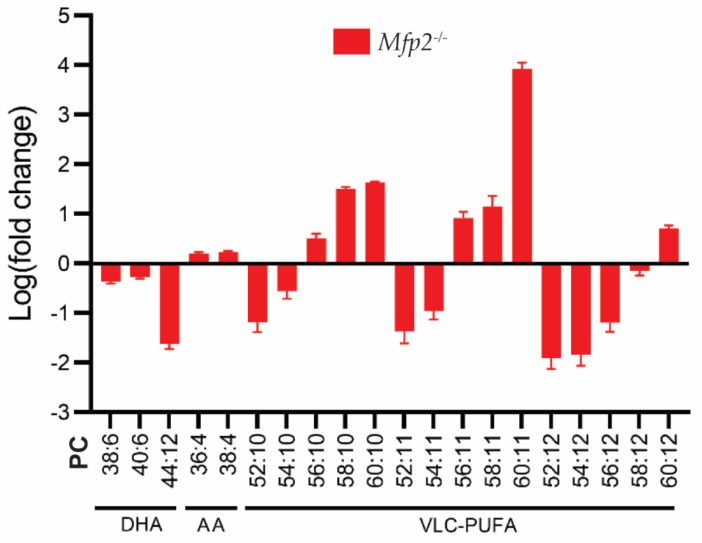
PUFA profile of *Mfp2^−/−^* neural retinas. Lipidome analysis on *Mfp2^−/−^* neural retinas revealed a severe decrease in DHA- and an increase in AA-containing PC phospholipids. The PC phospholipids containing VLC-PUFAs show a peculiar profile, as phospholipids containing ≤56 carbons in their side chains are severely reduced, while those containing >56 carbons accumulate. These PC species contain a VLC-PUFA and DHA moiety in the *sn*-1 and *sn*-2 position, respectively. Data are presented as log transformation of relative values to WT values.

With respect to the considerations above, we assessed the PUFA status of the *Mfp2^−/−^* retinas in more detail (Figure 4) [23]. Firstly, we found an overall decrease in DHA-containing phospholipid species, with a striking virtual depletion of those containing two DHA moieties (i.e., PC(44:12)). Of note, these decreases were accompanied by a compensatory increase in phospholipids containing *n*-6 PUFAs (such as arachidonic acid). Secondly, it was shown that phospholipids with ≥52 carbons and 10-12 double bonds in their side chains are composed of a tetra-, penta- or hexaenoic VLC-PUFA, respectively, in the *sn*-1 position and DHA in the *sn*-2 position. These phospholipid species displayed a peculiar profile. More specifically, phospholipids containing VLC-PUFAs with ≤34 carbons in their side chains were severely reduced, while those containing >34 carbons accumulated.

Keeping these observations in mind, two pertinent questions emerged: (i) Are these changes caused by a systemic or a local defect in peroxisomal β-oxidation? (ii) How do these changes in retinal lipidome relate to the observed phenotype?

#### 6.3.4. The Origin of the Altered PUFA Profile

It is generally accepted that the majority of the retinal DHA content is delivered by the systemic circulation [128]. Interestingly, the *Mfp2^−/−^* mice suffered from a hampered retinal supply, as evidenced by a decrease in DHA-containing phospholipids in plasma [23]. This could be due to impaired DHA synthesis in the liver, but also originate from a severely impaired bile acid synthesis and steatorrhea during the lactation period, resulting in a decreased intestinal lipid uptake [102]. In addition, the *Mfp2^−/−^* retinas showed suppressed levels of Adiponectin receptor 1 (ADIPOR1), a protein involved in retinal DHA retention and traffic, suggesting hampered retinal handling [23,151]. Interesting to note is that transcripts of the transporter Major facilitator superfamily domain-containing protein 2a (MFSD2a) were not significantly reduced in *Mfp2^−/−^* mice [23]. Like ADIPOR1, MFSD2a is an essential contributor to maintain appropriate retinal DHA levels [152,153].

In contrast, it is by far less clear what the local role of peroxisomal β-oxidation in the retina encompasses. It could be involved in the synthesis of DHA in photoreceptors and RPE cells, although it is generally accepted that the yield and rate are rather limited to meet the needs for POS synthesis [95,96,97,98]. We hypothesize that there is a clear distinction in its role in the two cell types. In photoreceptors, peroxisomal β-oxidation could be involved in the retroconversion of DHA into EPA, needed for the synthesis of VLC-PUFAs as mentioned before [151]. The reduced levels of VLC-PUFAs with ≤34 carbons in the *Mfp2^−/−^* neural retina could thus be due to lower levels of DHA and/or EPA. An alternative task of peroxisomal β-oxidation could be to counter excessive PUFA elongation in the ER, in order to establish a balance between synthesis and degradation, and to maintain appropriate relative VLC-PUFA levels. The accumulation of VLC-PUFAs with >C34 carbons in the *Mfp2^−/−^* neural retina is in line with the latter hypothesis and is likely the result of an uncountered elongation [23].

In the RPE, on the other hand, the potential role of peroxisomal β-oxidation seems more straightforward as these cells are exposed to a high PUFA load following the daily phagocytosis of shed POS [8,9]. It is generally accepted that these PUFAs are mostly recycled [128]. This is underscored by the fact that *n*-3 deficient rats exhibited similar levels of retinal DHA compared to control rats, highlighting the avid conservation of DHA in these circumstances [121]. It is, however, plausible that some PUFAs need to be degraded, which requires peroxisomal β-oxidation for fatty acids with ≥C22. In this respect, it was shown that DHA, originating from ingested POS, can be used by RPE cells for the synthesis of ketone bodies that subsequently mainly serve as energy source for photoreceptors [154].

#### 6.3.5. Link between the Altered PUFA Profile and Retinal Phenotype

The question remains whether the peculiar PUFA profile underlies the observed retinal abnormalities. To date, the exact function of retinal DHA and VLC-PUFAs is still under debate, but several lines of evidence demonstrate that they (directly) interact with the phototransduction process and that they have a role in photoreceptor development and survival [128,155]. In view of the latter, it is interesting that DHA and VLC-PUFAs can be converted into cytoprotective and anti-inflammatory lipid mediators in case of uncompensated retinal stress, such as neuroprotection D1 and elovanoids, respectively [156,157]. In view of the substrate shortage in the retina, it can be speculated that this leads to decreased synthesis of these mediators, possibly contributing to the retinopathy.

Moreover, valuable information can be obtained by comparing the retinal pathology of the *Mfp2^−/−^* mice with that of models with genetic ablation of proteins involved in retinal PUFA homeostasis**.** These proteins are involved in retinal DHA uptake and traffic (i.e., ADIPOR1 [151] and MFSD2a) [152,153] and membrane-type frizzled-related protein (MFRP) [158]) and incorporation of DHA into phospholipids (lysophosphatidic acid acyltransferase 3 (LPAAT3) [159]). The retinas of the corresponding mouse models display severe reductions of phospholipids containing DHA and VLC-PUFAs, with a compensatory increase in those containing AA. Overall, frequently observed defects include decreased retinal function, POS length reduction (i.e., impaired photoreceptor maturation) and progressive photoreceptor loss (i.e., impaired photoreceptor survival). However, there is large variability between these models. Even two knockout models of the same gene (*Mfsd2a*) display a diverging phenotype despite comparable changes in PUFA levels [152,153]. This variability makes it challenging to clearly determine the link between the observed deregulated PUFA profile and retinal pathologies. Still, considering the similarity with the retinal pathology of *Mfp2^−/−^* mice, it is highly likely that the alterations in the retinal PUFA levels play a crucial role.

## 7. Conclusions and Future Prospects

Both data from patients and mouse models indicate that proper peroxisomal function is essential to maintain retinal homeostasis, but the underlying mechanisms still remain largely unresolved.

By analysing diseases in which only a single peroxisomal protein was defective, leading to specific metabolic profiles, it became clear that saturated VLCFA accumulation does not cause discernible retinal damage, the effect of ether lipid deficiency is currently unclear and the accumulation of branched chain fatty acids causes significant retinal pathology.

Furthermore, it is highly likely that a disturbed retinal PUFA homeostasis strongly contributes to the retinopathy observed in ZSD and peroxisomal β-oxidation (e.g., ACOX1 and MFP2) deficiency patients. Unfortunately, no hard evidence is available from patients, but the severe PUFA deregulation coinciding with the early-onset retinal pathology in the *Mfp2^−/−^* mice supports this statement. It requires, however, further research to unravel (i) the exact local role of peroxisomal β-oxidation for retinal PUFA homeostasis, (ii) the contribution of systemic versus local peroxisomal β-oxidation deficiency to the retinal phenotype, and (iii) which cell type drives the retinal pathology. Future investigations on cell type selective (photoreceptors and RPE) MFP2 knockout mice, including lipid analyses, will shed light on these open questions.

It is striking that the retinal pathology of the *Mfp2^−/−^* mice is more severe compared to the *Pex1* knock-in and ACBD5 knockout mice. It will be instructive to perform a thorough comparison of the lipid content and the retinal morphology of these mouse models.

Although we focused in this review on the role of peroxisomes in lipid metabolism, other important functions cannot be excluded. For example, they might have an important role in the retinal antioxidant system, which is illustrated by the high expression of the antioxidant enzyme catalase in the RPE [12,13,14,15,16,17,18]. This is not surprising as these cells are constantly challenged by ROS stress due to their very active metabolism and exposure to high ambient oxygen partial pressures [8,160]. Furthermore, a possible role of peroxisomes in ciliogenesis has recently been postulated by several laboratories. Several lines of evidence were provided: (i) knockdown of *PEX* genes partially impaired ciliogenesis, (ii) a serine-threonine kinase, NDR2, which is essential in ciliogenesis, localizes to peroxisomes, (iii) peroxisomes mediate trafficking of cholesterol into ciliary membranes [161,162]. Interestingly, the POS are a form of primary cilia that also contain a large amount of cholesterol in the plasma membrane and in the membranes of discs at the base [163]. It could be inferred that also via these roles in ciliogenesis peroxisomes are important in the retina.

One major drawback in the ongoing retinal research in peroxisomal disorders is the lack of reports on histopathological changes and lipid content in (mild) patients, which makes it challenging to translate the findings from the available mouse models to the human situation. Nevertheless, these models are useful to elucidate the underlying mechanisms, which should drive the development of future therapies to combat these blinding diseases.

## Figures and Tables

**Table 1:** Overview of the biochemical abnormalities and ocular phenotypes in peroxisomal biogenesis disorders and single enzyme deficiencies.

Group	Peroxisomal Disorder	Biochemical Abnormalities						Ocular Phenotype
		VLCFA	DHA	PRIS	PHYT	D/THCA	PL	
Peroxisome biogenesis disorders	Zellweger spectrum disorders ^1^ [4,26,28,29,30,31,33,37,40,41]	↑	↓	↑	↑	↑	↓	- varied pigmentary retinopathy, including retinitis pigmentosa - optic atrophy, cataract, glaucoma and nystagmus
	RCDP type 1 and 5 [3,26]	-	-	-	-	-	↓	- retina is spared - cataract
Single enzyme deficiencies	Refsum disease [49]	-	-	-	↑	-	-	- retinitis pigmentosa is the cardinal symptom - miosis, attenuated pupillary light responses, iris atrophy and cataract
	X-ALD [50,51,52,53,54]	↑	-	-	-	-	-	- retina is spared - decreased visual acuity due to extensive brain lesions and demyelination of visual tract
	ABCD3 deficiency [55]	-	-	-	-	↑	-	- no ocular symptoms
	AMACR deficiency [56,57,58,59,60,61,62,63,64]	-	-	↑	(↑)	↑	-	- retinitis pigmentosa - optic atrophy, cataract and visual field defects
	ACOX1 deficiency [65,66,67,68,69,70,71,72]	↑	NK	-	-	-	-	- pigmentary retinopathy - optic atrophy
	ACOX2 deficiency [73,74,75]	-	-	-	-	↑	-	- no ocular symptoms
	MFP2 deficiency ^2^ [76,77,78,79]	↑	↓	↑	↑	↑	-	- pigmentary retinopathy - optic atrophy, cataract
	SCPx deficiency [80]	-	-	↑	-	(↑)	-	- no ocular symptoms
	ACBD5 deficiency [81,82,83]	↑	-	-	-	-	-	- cone-rod dystrophy and retinal pigmentary changes - optic atrophy
	RCDP type 2, 3 and 4 [84,85]	-	-	-	-	-	↓	- retina is spared - cataract

ABCD, ATP-binding cassette type D; ACBD5, Acyl-CoA binding domain-containing protein 5; ACOX, Acyl-CoA oxidase; AMACR, α-methylacyl-CoA racemase; DHA, docosahexaenoic acid; DHCA and THCA, di- and trihydroxycholestanoic acid; MFP2, multi-functional protein 2; PHYT, phytanic acid; PL, plasmalogens; PRIS, pristanic acid; RCDP, rhizomelic chondrodysplasia punctata; SCPx, sterol carrier protein x; VLCFA, very long chain fatty acid; X-ALD, X-linked adrenoleukodystrophy. -, normal; ↑, elevated; ↓, decreased; NK, not known. ^1^ Biochemical properties in Heimler syndrome patients are usually within normal limits, with occasional slight elevations in VLCFAs. DHA levels were not reported. ^2^ In a few mild MFP2 deficient patients, the metabolic parameters were (near) normal.

## Data Availability

Data is contained within the article.
